# Microneedling Combined With Topical Poly-L-Lactic Acid and Hyaluronic Acid for Skin Elasticity and Hydration: A Pilot Comparative Study

**DOI:** 10.7759/cureus.115756

**Published:** 2026-09-04

**Authors:** Victoria Arutinova, Nino Nishnianidze, Murat Tsintsadze, Anna Kadzhaya, George Sulamanidze

**Affiliations:** 1 Dermatology, School of Natural Sciences and Medicine, Ilia State University, Tbilisi, GEO; 2 Dermatology, Total Charm Orbeliani, Tbilisi, GEO; 3 Plastic Surgery, Total Charm Orbeliani, Tbilisi, GEO; 4 Scientific and Clinical Development, Aptos LLC, Tbilisi, GEO; 5 Plastic Surgery, Aptos LLC, Tbilisi, GEO

**Keywords:** biostimulation, combination therapy, microneedling, neocollagenesis, poly-l-lactic acid-hyaluronic acid

## Abstract

Background

Microneedling is a minimally invasive skin rejuvenation technique that can improve photoaging-related skin texture, fine lines, and overall skin quality. This exploratory pilot study compared preliminary effects and short-term tolerability of microneedling combined with a topical poly-L-lactic acid-hyaluronic acid (PLLA-HA) formulation versus microneedling alone for skin quality and aesthetic appearance.

Methods

Thirty healthy participants (mean age ~38 years) received three treatment sessions of either microneedling alone (anatomically adjusted needle depth up to 2 mm) or microneedling plus topical PLLA-HA at two-week intervals. Skin biophysical measurements were recorded at baseline and weeks 2, 4, 6, 8, and 12 using the Cutometer for elasticity parameters (R2, R5, R7) and the Corneometer for hydration. Additional outcomes included 3D imaging with LifeViz® Mini Pro (QuantifiCare SA, Biot, France), FACE-Q™ Satisfaction with Outcome and FACE-Q™ Satisfaction with Skin, and Global Aesthetic Improvement Scale (GAIS) scores.

Results

Baseline-adjusted analyses favored the combination group for all elasticity parameters and hydration compared with microneedling alone. By week 12, 3D imaging revealed greater improvements in skin evenness, wrinkles, pore visibility, and image-derived oiliness. FACE-Q Satisfaction with Skin showed little change, whereas Satisfaction with Outcome favored the combination group at week 12 (mean difference, 30.6 points; 95% CI, 23.8-37.4); GAIS also favored the combination group. Adverse events were limited to two cases of mild, transient treatment-site erythema.

Conclusion

Microneedling combined with topical PLLA-HA is safe and more effective than microneedling alone, providing superior improvements in skin elasticity, hydration, and overall aesthetic appearance, supporting the benefit of combining mechanical and topical biostimulatory approaches in facial rejuvenation. This pilot study had a small sample size, and potential selection and assessment bias due to lack of randomization, allocation concealment, and assessor blinding. The 12-week follow-up period may not fully capture the long-term biostimulatory effects of PLLA. Hence, larger, well-controlled studies with extended follow-ups are warranted.

## Introduction

Skin aging is associated with progressive reductions in elasticity, hydration, and structural support, leading to wrinkles, uneven texture, and laxity [1]. Microneedling is a minimally invasive rejuvenation technique that creates controlled microinjury, activates wound-healing pathways, and promotes dermal remodeling [2]. Clinical studies and systematic reviews demonstrate that microneedling can improve photoaging-related skin texture, fine lines, and overall skin quality, with a favorable safety profile [3]. In addition, microneedling can enhance transdermal delivery by creating temporary microchannels across the stratum corneum, potentially increasing penetration of topical agents [2].

Hyaluronic acid (HA) is widely used in aesthetic dermatology because of its hydrating, humectant, and extracellular matrix-supporting properties [4]. Clinical studies indicate that non-cross-linked HA formulations improve skin hydration and perceived skin quality [4]. Poly-L-lactic acid (PLLA) is a collagen-stimulating biomaterial that induces fibroblast activation and neocollagenesis, resulting in progressive and sustained improvements in skin quality over time [5-7]. Combining microneedling with a topical PLLA-HA formulation may therefore provide complementary effects: microneedling may facilitate delivery and initiate remodeling, HA may support early hydration and surface quality, and PLLA may contribute to longer term dermal restructuring.

The use of microneedling as a delivery-enhancing platform has been explored with growth factors, peptides, and other bioactive compounds [8,9]. PLLA has also been evaluated in combination with fractional microneedle radiofrequency [10], although this approach differs from topical PLLA-HA delivery with conventional microneedling. Comparative clinical evidence on topical PLLA-HA delivered in conjunction with conventional microneedling remains limited, and the relative contribution of the combination versus microneedling alone is not well established. Accordingly, this exploratory pilot study aimed to estimate the preliminary comparative effects of microneedling combined with PLLA-HA versus microneedling alone on facial skin quality. The PLLA-HA formulation was evaluated as a combined intervention, and the study was not designed to determine the individual contribution of PLLA or HA. Cutometer-derived R2, R5, and R7 (elasticity parameters) and Corneometer-derived hydration were the primary outcomes, while FACE-Q [11,12] and Global Aesthetic Improvement Scale (GAIS) [13,14] scores, and imaging-derived measures were secondary exploratory outcomes. A recent case series reported improvements in facial pore appearance and skin texture following microneedling combined with topical poly-D,L-lactic acid (PDLLA), although the specific contribution of the topical polymer could not be separated from the effects of microneedling alone [15].

## Materials and methods

This prospective, investigator-initiated exploratory scientific research study was conducted at the Dermatology department of the Total Charm Orbeliani Clinic (Tbilisi, Georgia) from October 2025 to March 2026. The study was designed to compare microneedling alone with microneedling combined with PLLA-HA and to estimate preliminary between-group differences in skin-quality outcomes. The primary outcomes were changes in Cutometer-derived skin elasticity parameters (R2, R5, and R7) and Corneometer-derived skin hydration from baseline to week 12. FACE-Q and GAIS scores, and imaging-derived skin-quality measures were considered secondary exploratory outcomes.

Ethical statement

The study was conducted in accordance with the ethical principles of the Declaration of Helsinki and Good Clinical Practice guidelines. All participants provided written informed consent. The study was approved by the Local Independent Ethics Committee at Total Charm LLC (approval no. 09-001; September 1, 2025) before participant recruitment began. The ethics committee was institutionally independent from Aptos LLC (Tbilisi, Georgia), the manufacturer of the investigated product, and Aptos LLC had no role in the ethics review or approval process. The approved protocol specifically included the PLLA-HA delivery method used in this study.

Study design

A clinic-based, non-probability sample of 30 healthy adults aged 22-50 years, with investigator-assessed reductions in facial skin elasticity and hydration, was enrolled. Participants were screened and enrolled consecutively by a non-treating investigator who was not aware of the upcoming treatment assignment; eligibility and written informed consent were finalized before referral to the treating investigator, who subsequently assigned participants according to the predefined alternating 1:1 sequence, resulting in 15 participants in the microneedling-only group and 15 participants in the microneedling plus PLLA-HA group. Participants received three treatment sessions at two-week intervals at weeks 0, 2, and 4. Assessments were performed at baseline before the first treatment and at weeks 2, 4, 6, 8, and 12; at weeks 2 and 4, all assessments were completed before the scheduled treatment. No formal hypothesis-driven sample-size calculation was performed because this was an exploratory pilot study; a pragmatic target of 30 participants, 15 per group, was selected to estimate outcome variability and preliminary comparative effects, estimate outcome variability, and generate effect-size estimates for a future randomized trial. Skin quality was assessed at each visit using cutometer-derived elasticity parameters, Corneometer-based hydration measurements, 3D imaging (LifeViz Mini Pro; QuantifiCare SA, Biot, France), GAIS assessments performed by both investigator and patients, and FACE-Q Satisfaction with Skin and FACE-Q Satisfaction with Outcome questionnaires. Safety was assessed at every visit by adverse event monitoring.

Eligibility Criteria

Inclusion criteria included age 22-50 years, general good health, clinically reduced facial skin elasticity and hydration as determined by investigator assessment before treatment allocation, willingness to complete all study visits, and agreement to avoid additional aesthetic procedures during follow-up. No prespecified quantitative Cutometer or Corneometer thresholds were used for eligibility; reduced elasticity was assessed clinically based on diminished skin recoil, while reduced hydration was based on clinical signs of dryness. Eligibility was determined before treatment allocation, and instrumental measurements were subsequently used for baseline characterization and longitudinal outcome assessment. Exclusion criteria were active dermatologic disease or infection in the treatment area, a history of keloid scarring, pregnancy or breastfeeding, unwillingness to use contraception when applicable, immunosuppression or recent immunosuppressive therapy, hypersensitivity to HA or PLLA, recent retinoid use, recent dermal filler treatment, or other recent skin-boosting procedures. A six-month washout period for previous cosmetic procedures and topical skin-boosting treatments was required. Potential confounding was reduced by standardized environmental conditions, fixed anatomical measurement sites, the same trained investigator for instrumental assessments, a 20-minute acclimatization period, avoidance of cosmetics on assessment days, and instructions to avoid other aesthetic treatments; residual confounding from baseline skin differences, home skincare, and sun exposure could not be fully excluded.

Interventions

Participants underwent three full-face microneedling sessions using Dermapen 4™ (DermapenWorld, Sydney, Australia), performed by the same trained physician at speed level 3. The handpiece was maintained perpendicular to the skin using consistent, even contact pressure. Passes were performed sequentially in vertical, horizontal, and oblique directions. Needle depth was adapted according to regional facial anatomy, with approximate guidance depths of 0.5 mm in the periorbital region, 0.9 mm on the forehead and temples, 1.5 mm on the chin and perioral region, and up to 2.0 mm on the cheeks. Two to four passes were performed according to tissue response, with uniform pinpoint bleeding used as the clinical treatment endpoint. Exact participant-level needle depths and pass numbers were not recorded. Before treatment, the face was cleansed, and topical lidocaine/prilocaine cream was applied. After removal of the anesthetic, the skin was re-cleansed with a 0.05% chlorhexidine solution immediately before microneedling. In the combination group, patients underwent microneedling with the PLLA-HA solution. For the PLLA-HA solution, Ellure HA (Aptos LLC) was used. Ellure HA is supplied in a sterile vial as lyophilized granules containing 200 mg of active components: 170 mg of poly-L-lactic acid and 30 mg of native non-cross-linked hyaluronic acid. The formulation was reconstituted immediately before treatment under aseptic conditions using sterile water for injection, with the volume selected according to clinically assessed skin thickness using the pinch method [16]: 10 mL for thick skin, 12 mL for medium skin, and 15 mL for thin or very thin skin. The reconstitution volume was recorded for each participant, corresponding to PLLA concentrations of 17.0, 14.2, and 11.3 mg/mL and HA concentrations of 3.0, 2.5, and 2.0 mg/mL, respectively. The reconstituted volume was divided equally and measured before application (5 + 5 mL, 6 + 6 mL, or 7.5 + 7.5 mL, according to the respective reconstitution volume), with half applied before microneedling and half immediately afterward, followed by gentle massage for five minutes. One complete vial was therefore used per treatment session, although the effective transdermal dose could not be quantified. The PLLA component consisted of spheroidal microparticles approximately 20-30 μm in size, with a molecular weight of approximately 60,000 Da and a narrow particle-size distribution; approximately 70% of particles had a smooth surface and 30% a porous surface. All study products originated from the same manufacturing batch. Because the formulation was present during microneedling, the procedure is more appropriately characterized as microneedling-assisted delivery rather than conventional topical application alone. The control group underwent the same microneedling protocol without PLLA-HA. Both groups received identical post-procedure instructions, including sunscreen, emollient use, and avoidance of irritants for 48 hours.

Outcome Measures

Skin hydration and skin elasticity/firmness were assessed noninvasively using corneometry and cutometry. All instrumental measurements and 3D image capture were performed by the same trained investigator at the study site under controlled environmental conditions. Participants underwent a 20-minute acclimatization period before assessment and were instructed to avoid application of topical cosmetics on the day of evaluation.

Stratum corneum hydration was measured with the Corneometer CM 825 (Courage + Khazaka Electronic GmbH, Cologne, Germany), a capacitance-based instrument that reports hydration in arbitrary units. Three measurements were obtained after cleansing at each standardized malar site, and the mean of the triplicate readings was used for analysis. The exact interval between cleansing and Corneometer measurement was not separately recorded. Skin elasticity was assessed using the Cutometer MPA 580 (Courage+Khazaka electronic GmbH). R2, R5, and R7 were dimensionless elasticity ratios expressed as percentages (ratio × 100), with higher values indicating greater elastic recovery. R2 represents overall viscoelastic recovery, R5 net elasticity (Ur/Ue), and R7 immediate elastic recovery relative to total deformation (Ur/Uf). Measurements were obtained using a 2-mm probe at a suction pressure of 450 mbar, with two-second suction and two-second relaxation phases. Three measurement cycles were performed, and the mean of the three cycles was used for analysis. Measurements were processed using MPA CTplus software, version 1.1.7.3 (Courage + Khazaka Electronic GmbH), with calibration performed according to the manufacturer’s routine procedure before the study. All instrumental measurements followed a reproducible protocol using fixed anatomical landmarks and controlled environmental conditions (temperature 20-22 °C; relative humidity 40%-60%). The measurement site was the midpoint between the lateral canthus and the alar-base line, as illustrated in Figure 1.

**Figure 1:**
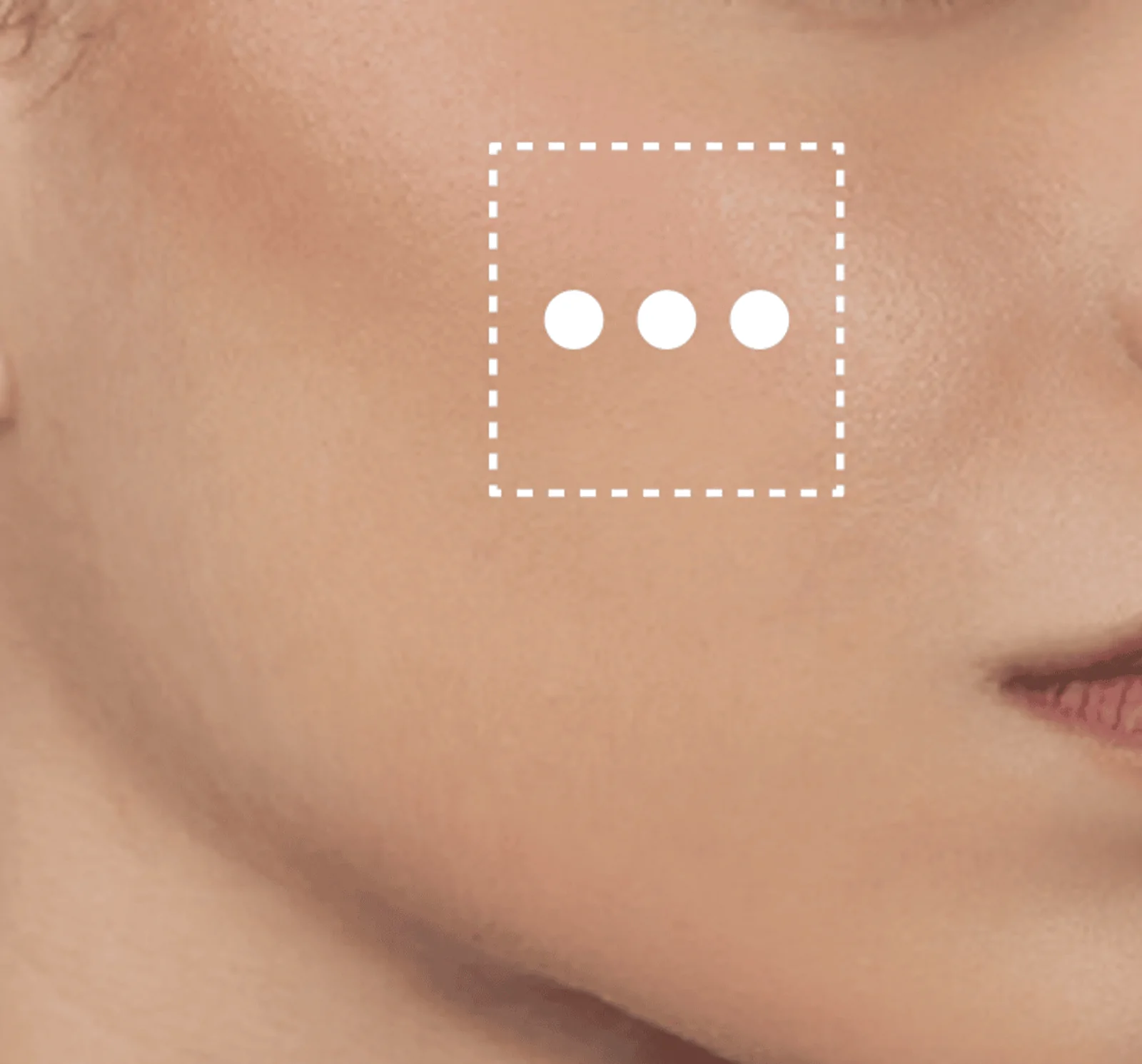
Standardized malar measurement site for Corneometer and Cutometer assessments

Patient-reported outcomes included scores on FACE-Q (Memorial Sloan Kettering Cancer Center, 2013), a validated instrument for evaluating patient satisfaction in aesthetic procedures [11,12]. The FACE-Q Satisfaction with Skin module consists of 12 items assessing patients’ perceptions of facial skin appearance, including smoothness, hydration, radiance, and overall quality, while the FACE-Q Satisfaction with Outcome module includes six items evaluating overall satisfaction with treatment results. Satisfaction with Skin was completed at baseline and at each follow-up visit, whereas Satisfaction with Outcome was completed only at follow-up visits.

Overall aesthetic improvement was assessed separately by the participant and the treating physician using the five-point GAIS [13,14]. Physician-rated GAIS scoring was performed by the non-treating investigator who was not involved in group allocation and remained blinded to treatment assignment. Scores were defined as 1, very much improved; 2, much improved; 3, improved; 4, no change; and 5, worsened. GAIS was completed at each follow-up visit. Wrinkles, pore visibility, skin evenness, and image-derived oiliness were evaluated separately on the right and left sides of the face using a three-dimensional stereophotogrammetry system (LifeViz Mini Pro, QuantifiCare, Skin Analysis software version 3.15.1.0). Imaging was performed under standardized lighting and positioning conditions according to the study acquisition protocol. At each visit, four standardized photographs were obtained and reconstructed into a single three-dimensional image. A predefined cheek region of interest was assessed on each side, with the same anatomical region used at every visit. Changes were expressed using the software-derived scale, with positive values indicating improvement from baseline. The oiliness parameter represented image-derived facial surface oiliness/shine rather than direct measurement of sebum production. The underlying parameter algorithms are proprietary and were not independently validated within this study.

Safety Assessment

Safety was monitored at every visit through participant reporting and physical examination of the treated area. Adverse events were graded by severity and assessed in relationship to treatment, with particular attention to erythema, edema, desquamation, discomfort, infection, and delayed reactions.

Statistical analysis

Descriptive statistics were generated using Microsoft® Excel (Microsoft Corporation, Redmond, USA), and inferential analyses were performed in R software, version 4.5.2 (R Foundation for Statistical Computing, Vienna, Austria). Continuous variables are reported as means ± standard deviations or median with first and third quartiles, as appropriate, and categorical variables as n (%).

The primary outcomes were changes in instrumental measures of skin elasticity obtained with the Cutometer (R2, R5, and R7) and skin hydration assessed using the Corneometer. FACE-Q scales, GAIS assessments, and imaging-derived skin-quality parameters were considered secondary exploratory outcomes. The primary assessment timepoint for the instrumental outcomes was week 12; measurements at weeks 2, 4, 6, and 8 were used to characterize the longitudinal treatment response.

The primary inferential analysis for repeated continuous outcomes used linear mixed-effects models to account for repeated measurements within the same patients over time. Treatment group, timepoint (weeks 2, 4, 6, 8, and 12), and the treatment-by-timepoint interaction were included as fixed effects, with patient ID included as a random effect to account for within-subject variability. Visit-specific between-group contrasts were estimated using estimated marginal means (emmeans). For ordinal outcomes, including GAIS, ordinal mixed models were applied using the same treatment group, timepoint, treatment-by-timepoint, and patient-level structure.

Mann-Whitney U tests were performed at individual timepoints as supportive analyses. Bonferroni correction was applied across the five post-baseline visits within each individual outcome. Thus, the multiplicity adjustment addressed repeated visit-specific comparisons within an outcome and was not applied globally across the different instrumental, FACE-Q, GAIS, and imaging outcome domains. Both unadjusted and Bonferroni-adjusted p-values were generated. Secondary outcomes were therefore interpreted as exploratory, with emphasis placed on estimated treatment effects and their uncertainty rather than statistical significance alone.

Imaging-derived skin-quality parameters were analyzed separately for the two facial sides. Cutometer and Corneometer measurements were obtained at a single predetermined anatomical site and therefore did not involve bilateral measurements. Within-participant correlation between right- and left-sided imaging measurements was not explicitly modeled; therefore, the bilateral imaging analyses were considered exploratory.

Because substantial baseline imbalances were observed, additional baseline-adjusted longitudinal linear mixed-effects models were performed for R2, R5, R7, and skin hydration. For each outcome, the post-baseline measurement was modeled as the dependent variable, with the corresponding baseline value, treatment group, timepoint, and treatment-by-timepoint interaction included as fixed effects and patient ID included as a random intercept. Visit-specific adjusted between-group effects were estimated and are reported with 95% confidence intervals.

Model assumptions were assessed using residual diagnostics and were considered acceptable. Missing observations were not imputed; analyses were based on the available observations for each outcome and visit.

## Results

A total of 30 participants were screened, enrolled, and allocated to two groups: 15 to microneedling alone and 15 to microneedling plus PLLA-HA. All 30 participants completed the 12-week study, although week-12 Cutometer and Corneometer measurements were unavailable for four participants: one in the microneedling-only group and three in the combination group. No participant discontinued because of an adverse event or treatment intolerance. Baseline demographic characteristics are summarized in Table 1; the cohort consisted of predominantly female participants, with 14 (93.3%) women and 1 (6.7%) man in each group. The mean age was 37-38 years.

**Table 1:** Baseline participant demographics SD, standard deviation; Q1, first quartile; Q3, third quartile; PLLA, poly-L-lactic acid; HA, hyaluronic acid

Parameter/Group	Microneedling	Microneedling with PLLA-HA
Age		
Mean (SD), years	37.1 (5.9)	38.2 (6.2)
Median (Q1; Q3), years	38 (33; 41.5)	40 (33.5; 43)
Minimum-maximum, years	24-44	27-47
Sex		
Males, %	1 (6.7%)	1 (6.7%)
Females, %	14 (93.3%)	14 (93.3%)

Instrumental outcomes​

At baseline, mean ± standard deviation values in the microneedling-only group were 87.8 ± 6.4% for R2, 60.0 ± 7.5% for R5, and 57.9 ± 10.7% for R7; corresponding values in the microneedling plus PLLA-HA group were 67.9 ± 8.5%, 51.9 ± 8.7%, and 39.9 ± 8.4%, respectively. The microneedling-only group showed an early decrease followed by recovery toward baseline, whereas the combination group showed progressive improvement through week 12. At week 12, mean changes from baseline in the combination group were 15.0 ± 7.1% for R2, 20.7 ± 5.7% for R5, and 18.2 ± 4.2% for R7, corresponding to 22.0%, 39.8%, and 45.6% of the respective baseline means. Supportive change-from-baseline analyses favored the microneedling plus PLLA-HA group at each post-baseline visit; however, given the substantial baseline imbalance, additional baseline-adjusted longitudinal analyses were performed.

After adjustment for baseline values, the between-group differences continued to favor the microneedling plus PLLA-HA group throughout follow-up visits. For R2, the adjusted between-group differences (microneedling plus PLLA-HA minus microneedling alone) were 18.80 percentage points at week 2 (95% CI, 14.19-23.41), 14.07 at week 4 (95% CI, 9.46-18.68), 7.61 at week 6 (95% CI, 3.01-12.22), 12.06 at week 8 (95% CI, 7.45-16.67), and 7.76 at week 12 (95% CI, 3.05-12.47). For R5, the corresponding adjusted between-group differences were 15.86 percentage points at week 2 (95% CI, 12.27-19.46), 14.94 at week 4 (95% CI, 11.35-18.53), 18.61 at week 6 (95% CI, 15.02-22.20), 18.78 at week 8 (95% CI, 15.19-22.37), and 19.99 at week 12 (95% CI, 16.31-23.67). For R7, adjusted between-group differences were 13.26 percentage points at week 2 (95% CI, 8.25-18.27), 12.93 at week 4 (95% CI, 7.92-17.94), 11.56 at week 6 (95% CI, 6.55-16.57), 15.11 at week 8 (95% CI, 10.11-20.12), and 16.17 at week 12 (95% CI, 11.07-21.28). At the primary week-12 assessment, the adjusted between-group differences were 7.76 percentage points for R2 (95% CI, 3.05-12.47), 19.99 for R5 (95% CI, 16.31-23.67), and 16.17 for R7 (95% CI, 11.07-21.28). All adjusted 95% confidence intervals excluded zero, indicating that the between-group differences remained evident after accounting for baseline elasticity. The R2, R5, and R7 trajectories are shown in Figures 2, 3, 4, respectively.

**Figure 2:**
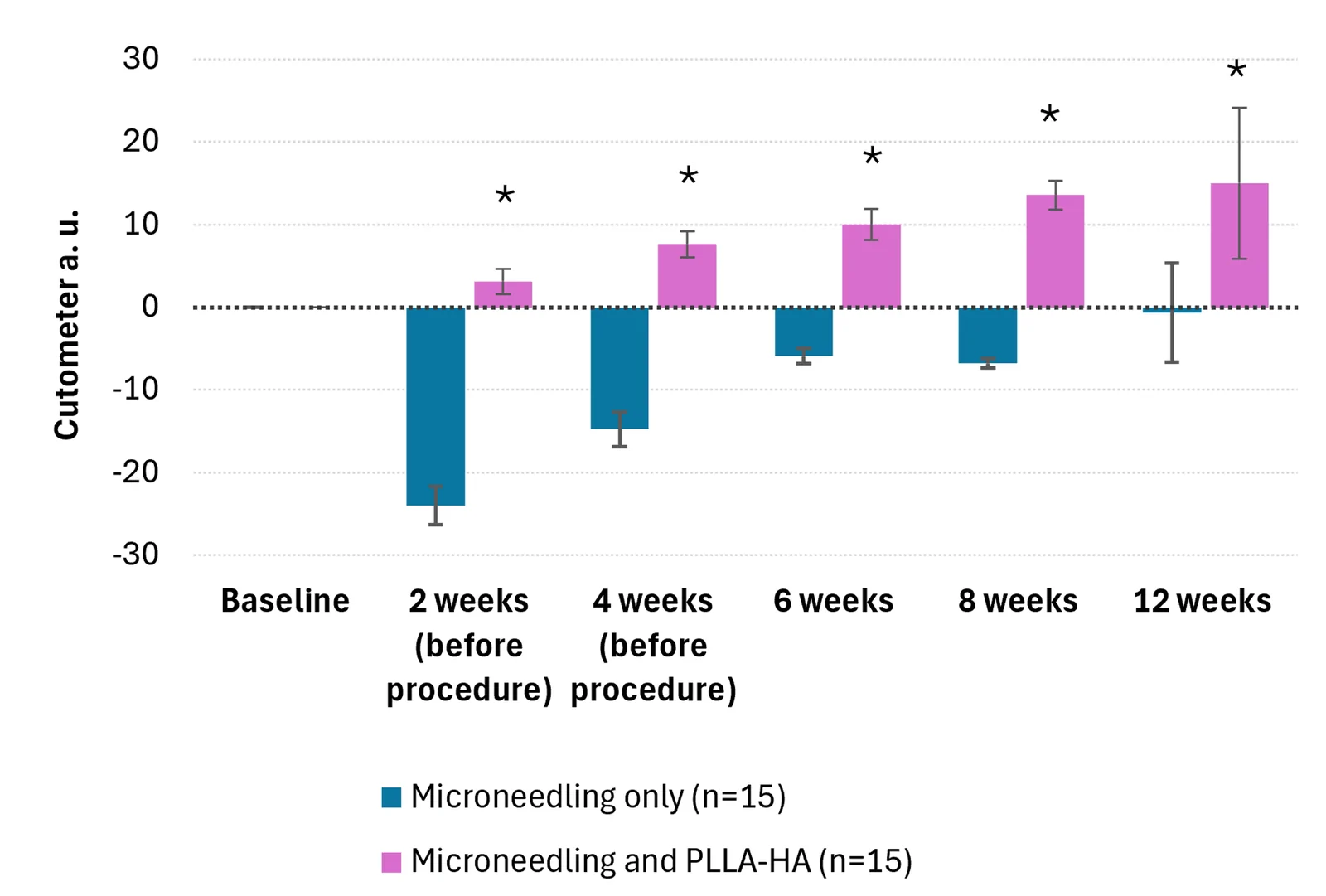
Changes in the R2 parameter (gross elasticity) over 12 weeks Values are means ± standard errors in arbitrary units. Microneedling-only group, n = 15; microneedling plus poly-L-lactic acid-hyaluronic acid (PLLA-HA) group, n = 15. Asterisks indicate p < 0.001 for the between-group difference in change from baseline at the corresponding visit, using the Mann-Whitney U test and linear mixed-effects model.

**Figure 3:**
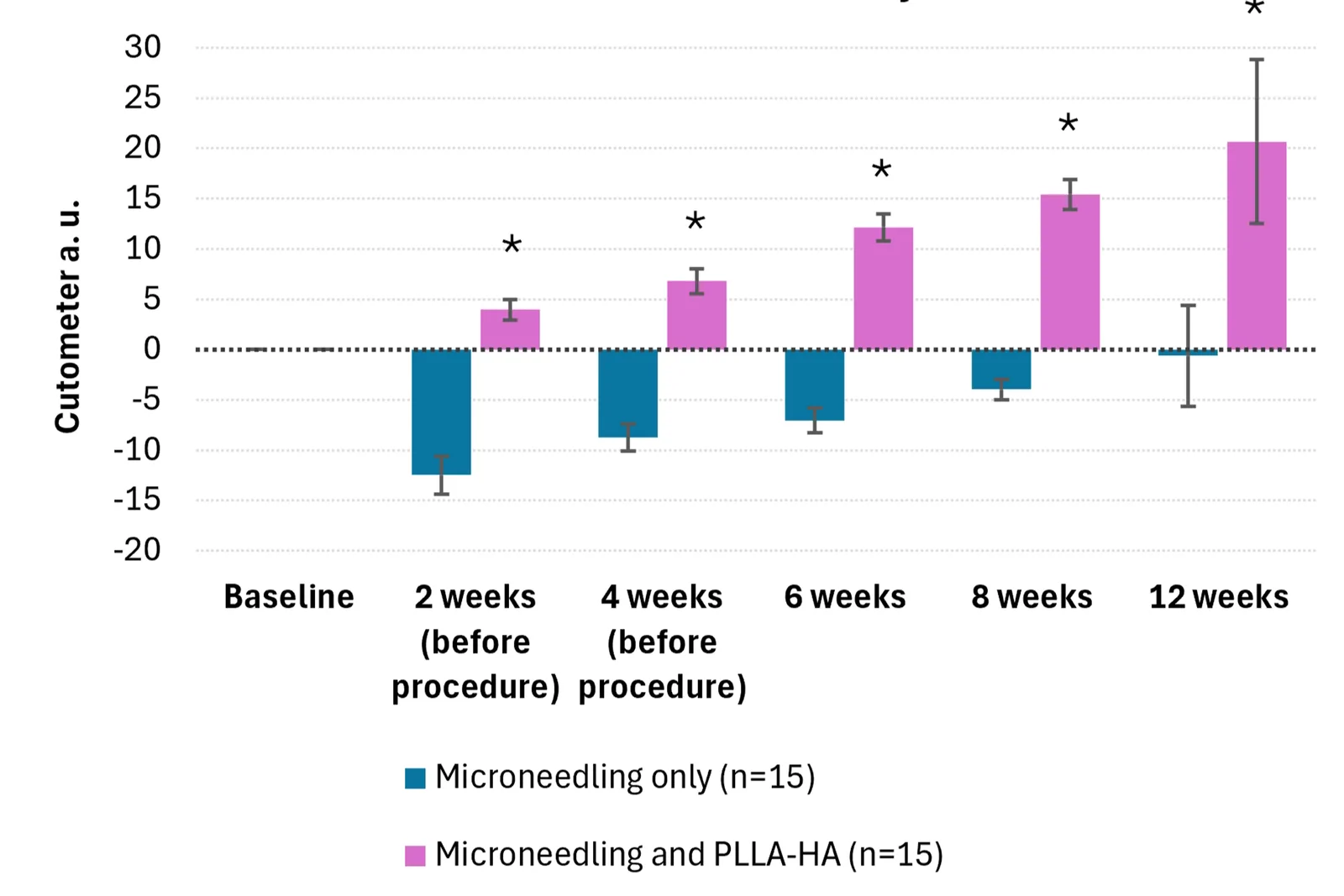
Changes in the R5 parameter (net elasticity) over 12 weeks Values are means ± standard errors in arbitrary units. Microneedling-only group, n = 15; microneedling plus poly-L-lactic acid-hyaluronic acid (PLLA-HA) group, n = 15. Asterisks indicate p < 0.001 for the between-group difference in change from baseline at the corresponding visit, using the Mann-Whitney U test and linear mixed-effects model. The prespecified threshold for statistical significance was two-sided p < 0.01.

**Figure 4:**
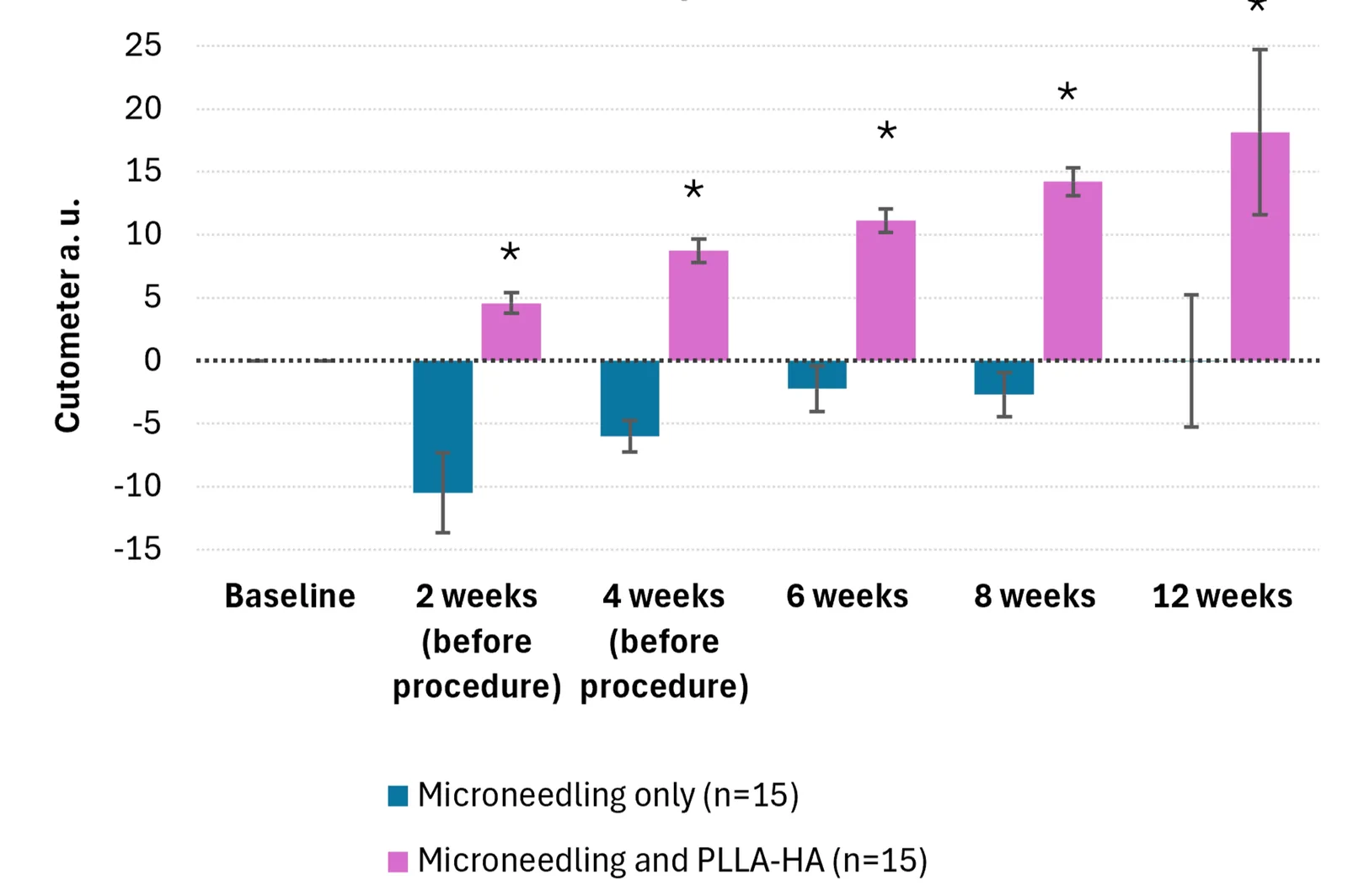
Changes in the R7 parameter (biological elasticity) over 12 weeks Values are means ± standard errors in arbitrary units. Microneedling-only group, n = 15; microneedling plus poly-L-lactic acid-hyaluronic acid (PLLA-HA) group, n = 15. Asterisks indicate p < 0.001 for the between-group difference in change from baseline at the corresponding visit, using the Mann-Whitney U test and linear mixed-effects model. The prespecified threshold for statistical significance was two-sided p < 0.01.

Skin hydration

Figure 5 shows the changes in skin hydration during the study, highlighting the distinct trends observed in the treatment groups. While the microneedling-only group began with a baseline hydration value of 56.5 ± 7.5, the combination group started at a baseline of 41.2 ± 10.7. The microneedling-only group experienced an initial decrease in hydration at two weeks, with a further gradual increase to baseline values by week 12. The microneedling plus PLLA-HA group showed a pronounced and consistent improvement from baseline throughout the study, resulting in an increase in skin hydration of 13.8 ± 13.9 (33.5% of initial values) by week 12. Supportive change-from-baseline analyses favored the microneedling plus PLLA-HA group at weeks 2, 4, and 12. Because baseline hydration differed substantially between groups, an additional baseline-adjusted linear mixed-effects analysis was performed. Post-baseline hydration was modeled with baseline hydration, treatment group, visit, and treatment-by-visit interaction as fixed effects and participant as a random effect. After adjustment for baseline hydration, the between-group differences favored the microneedling plus PLLA-HA group at weeks 2, 4, 6, 8, and 12, with adjusted differences of 22.21 (95% CI, 12.01-32.40), 21.99 (95% CI, 11.79-32.18), 20.00 (95% CI, 9.80-30.20), 14.70 (95% CI, 4.50-24.90), and 20.77 arbitrary units (95% CI, 10.51-31.03), respectively. At the primary week-12 assessment, the adjusted between-group difference was 20.77 arbitrary units (95% CI, 10.51-31.03). The treatment-by-visit interaction was significant (p<0.001).

**Figure 5:**
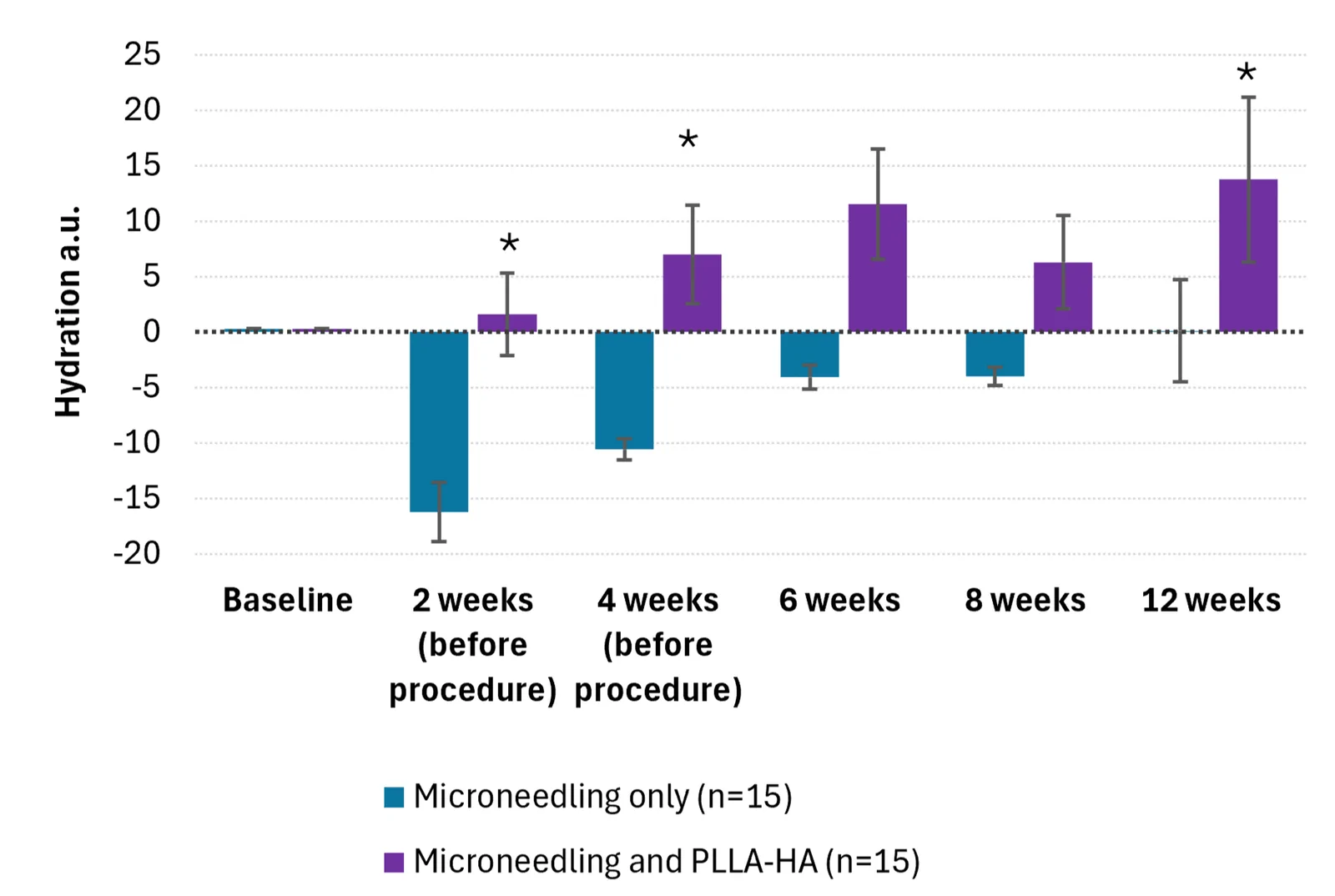
Skin hydration changes throughout the study (from baseline over 12 weeks) Values are mean ± standard error in arbitrary units. Microneedling-only group, n = 15; microneedling plus poly-L-lactic acid-hyaluronic acid (PLLA-HA) group, n = 15. Asterisks indicate p < 0.001 for the between-group difference at the corresponding visit, using the Mann-Whitney U test and linear mixed-effects model. The prespecified threshold for statistical significance was two-sided p < 0.01.

Patient-reported outcomes

Patient-reported outcomes assessed using the FACE-Q Satisfaction with Skin questionnaire are presented in Table 2.

**Table 2:** Total scores for FACE-Q Satisfaction with Skin and FACE-Q Satisfaction with Outcome at study visits Raw FACE-Q scores were converted to 0-100 transformed scores, using the validated Rasch conversion tables for the respective scales, with higher values indicating greater satisfaction. Satisfaction with Outcome was not administered at baseline. *p < 0.001 for the between-group difference at the corresponding visit, using the Mann-Whitney U test and linear mixed-effects model. The prespecified threshold for statistical significance was two-sided p < 0.01. M, microneedling alone; PLLA-HA, poly-L-lactic acid-hyaluronic acid; N/A, not applicable

Questionnaire	Satisfaction with Skin		Satisfaction with Outcome	
Group	M	M + PLLA-HA	M	M + PLLA-HA
Baseline	77.3 ± 4.7	81.6 ± 5.3	N/A	N/A
2 weeks	76.1 ± 2.1	83.1 ± 8.9	58.5 ± 5.3	92.6 ± 10.7*
4 weeks	77.3 ± 2.9	82.0 ± 8.3	54.9 ± 8.3	92.9 ± 8.3*
6 weeks	78.5 ± 5.7	80.8 ± 5.6	57.3 ± 9.0	92.3 ± 11.6*
8 weeks	78.5 ± 4.4	81.1 ± 5.8	59.2 ± 6.4	90.9 ± 11.7*
12 weeks	77.3 ± 4.5	82.5 ± 7.4	59.6 ± 7.3	90.2 ± 10.5*

FACE-Q Satisfaction with Skin scores were high at baseline in both groups and changed little over time, which likely limited the ability to detect improvement. No within-group change was statistically significant, and the between-group pattern did not show a consistent treatment-related increase.

FACE-Q Satisfaction with Outcome, which was administered only after treatment, represented post-treatment satisfaction rather than improvement from baseline and was consistently higher in the combination group throughout follow-up. At every post-treatment visit, the between-group difference favored microneedling plus PLLA-HA (p < 0.001; Table 2).

Item-level response distributions supported the total-score findings. From week 2 through week 12, the combination group had a larger proportion of “definitely agree” responses, whereas responses in the microneedling-only group were more dispersed across “somewhat agree” and disagreement categories. These distributions are shown in Figures 6-7.

**Figure 6:**
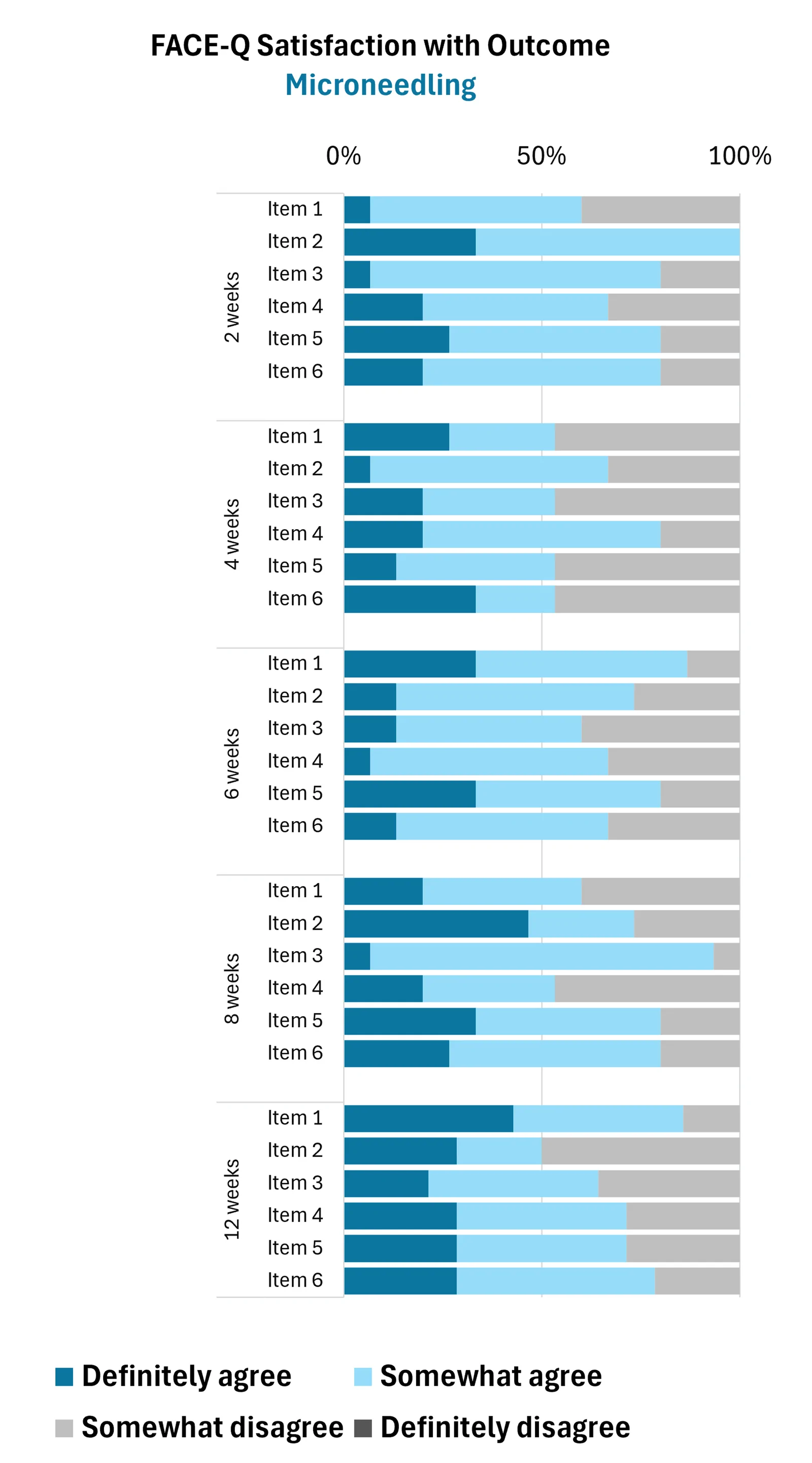
Distribution of responses to individual questions of the FACE-Q Satisfaction with Outcome questionnaire, by study subjects, for microneedling alone at weeks 2, 4, 6, 8, and 12 Response categories are shown as percentages of available responses. © 2013 Memorial Sloan-Kettering Cancer Center, Memorial Hospital for Cancer and Allied Diseases, Sloan-Kettering Institute for Cancer Research, Anne Klassen, and Stefan Cano. All rights reserved. FACE-Q® is a U.S. registered trademark of Memorial Sloan-Kettering Cancer Center, 1275 York Avenue, New York, NY 10065.

**Figure 7:**
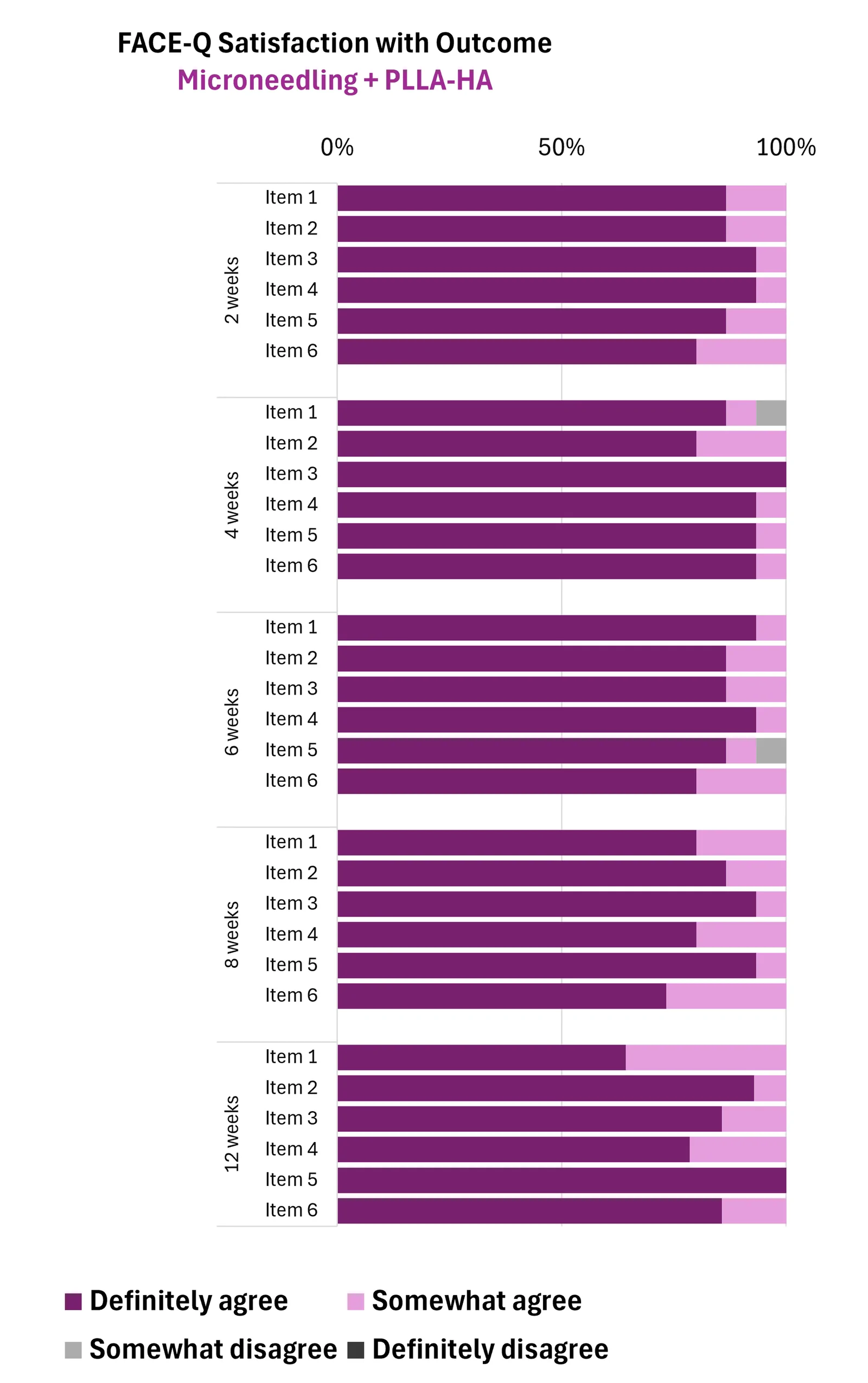
Distribution of responses to individual questions of the FACE-Q Satisfaction with Outcome questionnaire, by study subjects, for microneedling with PLLA-HA at weeks 2, 4, 6, 8, and 12 Response categories are shown as percentages of available responses. PLLA-HA, poly-L-lactic acid-hyaluronic acid © 2013 Memorial Sloan-Kettering Cancer Center, Memorial Hospital for Cancer and Allied Diseases, Sloan-Kettering Institute for Cancer Research, Anne Klassen, and Stefan Cano. All rights reserved. FACE-Q® is a U.S. registered trademark of Memorial Sloan-Kettering Cancer Center, 1275 York Avenue, New York, NY 10065.

The higher Satisfaction with Outcome scores indicate greater post-treatment satisfaction in the combination group; however, because participants were aware of treatment assignment, expectancy effects cannot be excluded, and this outcome should not be interpreted as independent evidence of comparative biological efficacy.

Global Aesthetic Improvement Scale

Patient- and physician-rated aesthetic improvement was assessed with the five-point GAIS. The distribution of GAIS grades at each visit is presented in Figure 8. No participant was rated as worsened at any time point. For patient-rated GAIS, improvement was reported by 14/15 (93.3%) participants in the microneedling-only group and 13/15 (86.7%) participants in the combination group at week 2; by 15/15 (100%) and 14/15 (93.3%), respectively, at week 4; by 14/15 (93.3%) and 15/15 (100%), respectively, at week 6; and by 9/15 (60.0%) and 15/15 (100%), respectively, at week 8. At week 12, one participant in each group had missing patient-rated GAIS data; therefore, the denominator was 14 participants per group. Improvement was reported by 7/14 (50.0%) participants in the microneedling-only group and 14/14 (100%) participants in the combination group. No imputation was performed. Between-group differences in patient-rated GAIS were statistically significant from weeks 6 through 12 (p < 0.01, Mann-Whitney U test).

**Figure 8:**
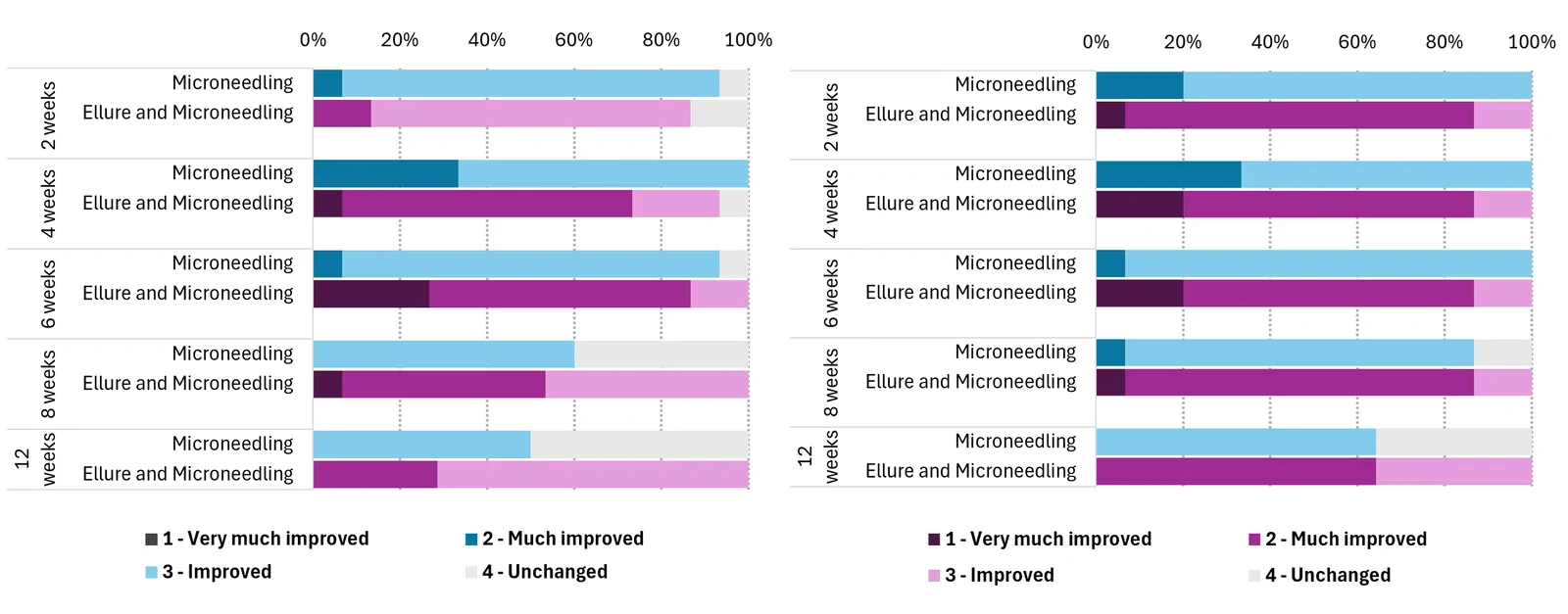
Patient-rated (left) and physician-rated (right) GAIS, shown as the percentage of available participants in each grade Global Aesthetic Improvement Scale (GAIS) score: 1, very much improved; 2, much improved; 3, improved; 4, unchanged; 5, worsened. Grade 5 is not displayed because no participant was rated as worsened. Between-group comparisons were done using the Mann-Whitney U test and ordinal mixed-effects model; statistical significance was defined as two-sided p < 0.01.

For physician-rated GAIS, improvement was reported in 15/15 (100%) participants in both groups at weeks 2, 4, and 6. At week 8, improvement was reported in 13/15 (86.7%) participants in the microneedling-only group and 15/15 (100%) participants in the combination group. At week 12, one participant in each group had missing physician-rated GAIS data. Improvement was reported in 9/14 (64.3%) participants in the microneedling-only group and 14/14 (100%) participants in the combination group; 5/14 (35.7%) participants in the microneedling-only group were rated as unchanged. No participant was rated as worsened. Between-group differences in physician-rated GAIS scores were statistically significant at all follow-up visits (p < 0.01, Mann-Whitney U test and ordinal mixed-effects model).

Patient- and investigator-rated GAIS showed similar directional trends; however, patient ratings were unblinded, whereas physician-rated GAIS scoring was performed by an allocation-blinded non-treating investigator.

Skin quality outcomes

Across all QuantifiCare parameters assessed (wrinkles, pores, evenness, and image-derived oiliness), both treatment groups demonstrated improvement over time; however, the combination group consistently showed statistically significantly greater magnitude and durability of effect when compared to microneedling alone. The observed change from baseline in the microneedling + PLLA-HA group was statistically significantly higher than in the microneedling-only group at all time points, except for evenness at week 6, where statistical significance was not reached.

Wrinkles

Wrinkle scores improved on both sides of the face by week 2 and peaked around week 4. The combination group maintained greater improvement than the microneedling-only group at all follow-up visits, with statistically significant between-group differences on both sides (p < 0.001, in the Mann-Whitney U tests and mixed-effects models). Right- and left-sided results are shown in Figure 9.

**Figure 9:**
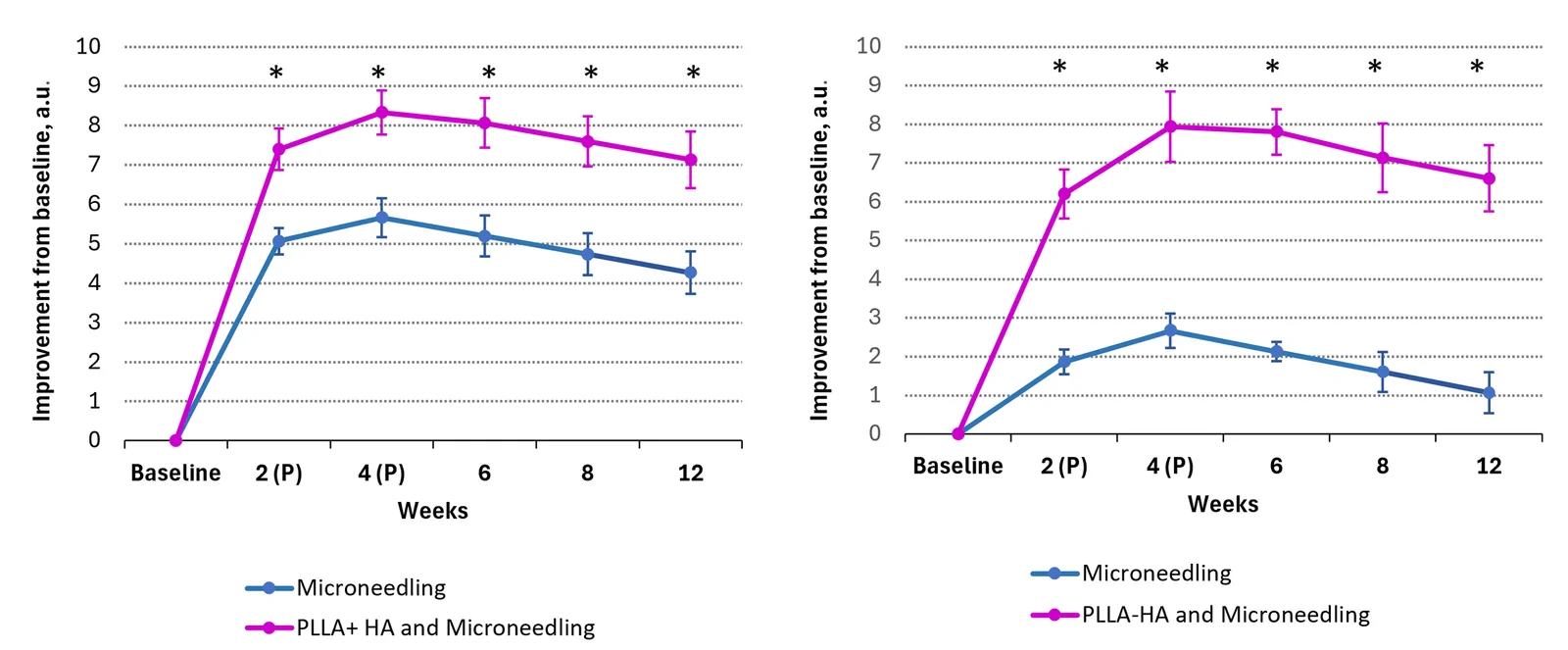
Wrinkle severity improvement, from baseline, for the right side of the face (left) and the left side of the face (right) Values are means ± standard errors in arbitrary units. Asterisks indicate p < 0.001 for the between-group difference, using the Mann-Whitney U test and linear mixed-effects model. Statistical significance was defined as two-sided p < 0.01. PLLA-HA, poly-L-lactic acid-hyaluronic acid; P, treatment procedure performed at that visit

Pore Visibility

Pore visibility improved on both sides by week 2 and peaked around week 4. The combination group showed larger improvements than microneedling alone at every follow-up visit, with statistically significant between-group differences on both sides (p < 0.001 in the Mann-Whitney U tests and mixed-effects models). The trajectories are presented in Figure 10.

**Figure 10:**
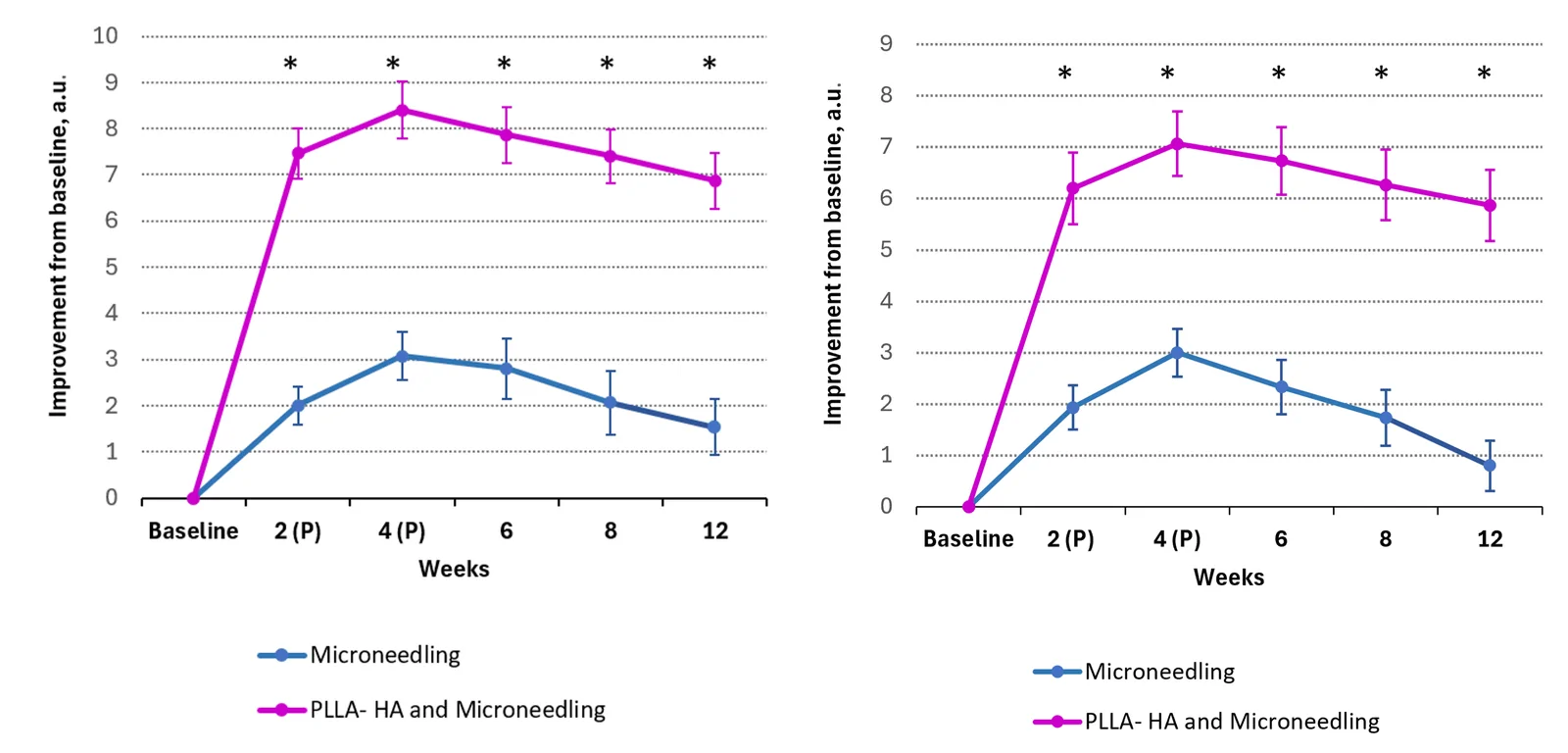
Pore visibility improvement, from baseline, for the right side of the face (left) and the left side of the face (right) Values are means ± standard errors in arbitrary units. Asterisks indicate p < 0.001 for the between-group difference, using the Mann-Whitney U test and linear mixed-effects model. Statistical significance was defined as two-sided p < 0.01. PLLA-HA, poly-L-lactic acid-hyaluronic acid; P, treatment procedure performed at that visit

Skin Evenness

The microneedling-only group showed modest, transient improvement in skin evenness, whereas the combination group showed larger changes that were better maintained through week 12. Between-group differences were statistically significant at most visits; the difference for right-sided evenness at week 6 was not statistically significant. Figure 11 presents the right- and left-sided trajectories.

**Figure 11:**
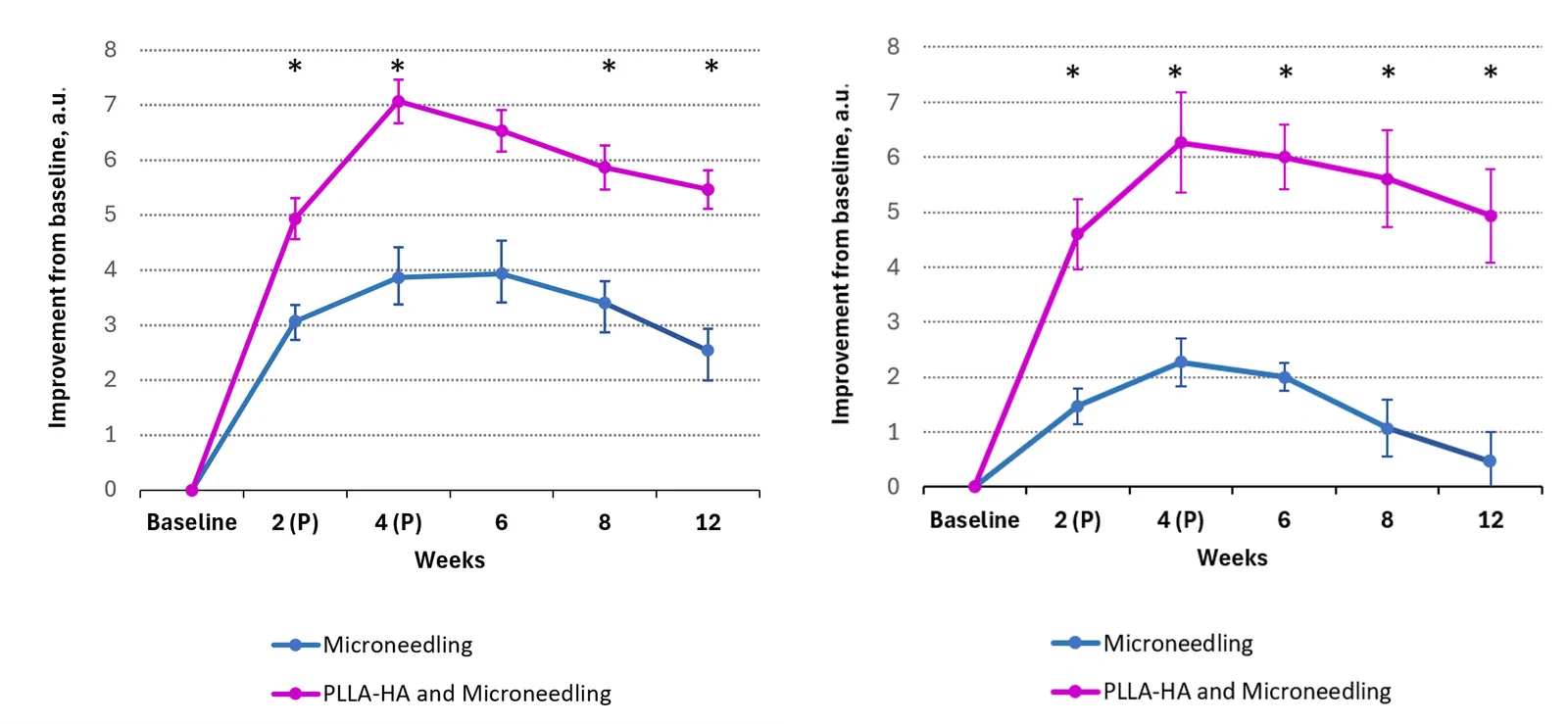
Skin evenness improvement for the right side of the face (left) and the left side of the face (right) Values are means ± standard errors in arbitrary units. Asterisks indicate p < 0.001 for the between-group difference, using the Mann-Whitney U test and linear mixed-effects model; the right-sided comparison at week 6 was not statistically significant. Statistical significance was defined as two-sided p < 0.01. PLLA-HA, poly-L-lactic acid-hyaluronic acid; P, treatment procedure performed at that visit

Image-Derived Skin Oiliness

Both groups showed reductions in image-derived skin oiliness, but the response was larger and more sustained in the combination group. Between-group differences were statistically significant at every post-baseline visit for both sides of the face (p < 0.001, in the Mann-Whitney U tests and mixed-effects models). The findings are shown in Figure 12.

**Figure 12:**
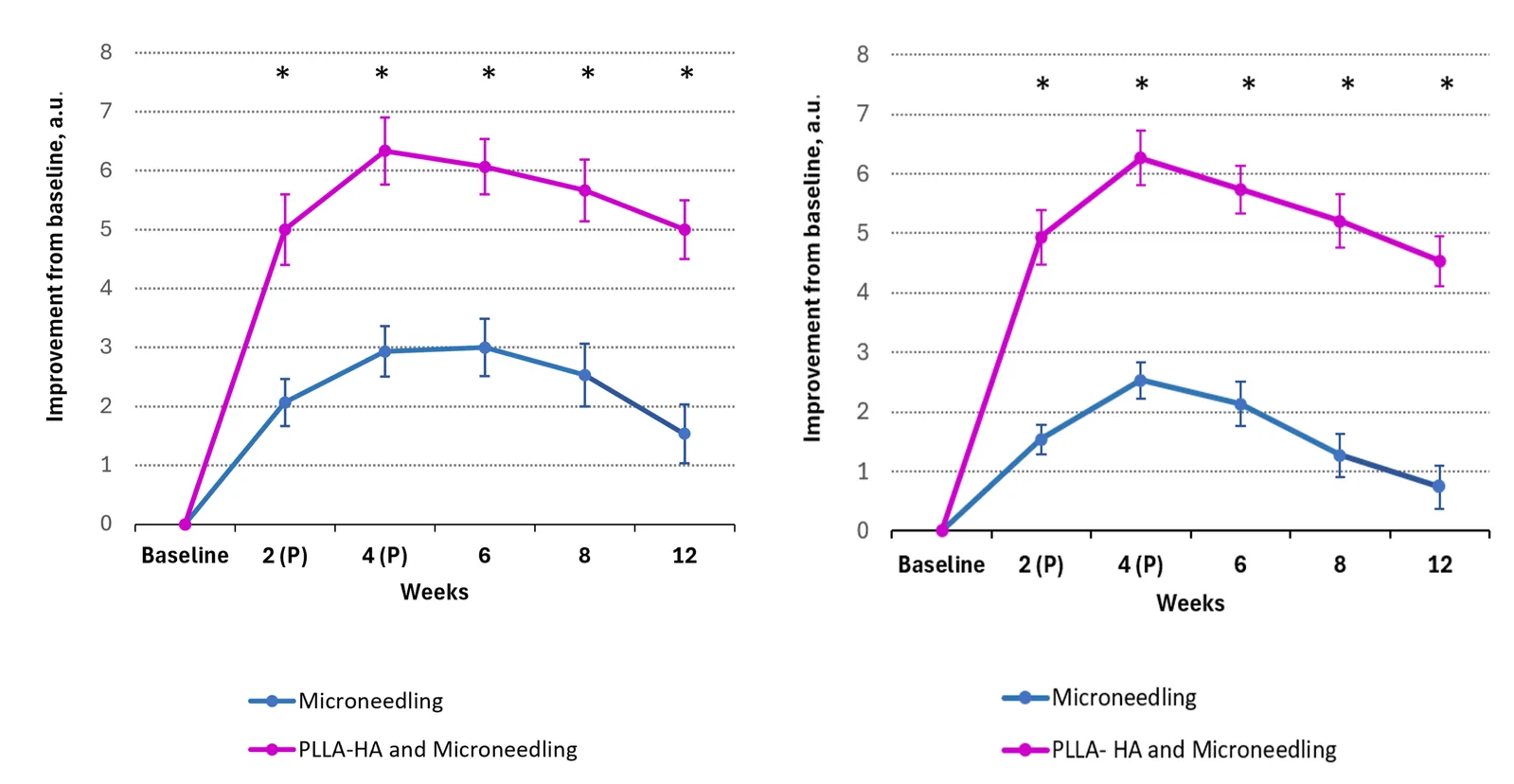
Image-derived skin oiliness improvement for the right side of the face (left) and the left side of the face (right) Values are means ± standard errors in arbitrary units. Asterisks indicate p < 0.001 for the between-group difference, using the Mann-Whitney U test and linear mixed-effects model. Statistical significance was defined as two-sided p < 0.01. PLLA-HA, poly-L-lactic acid-hyaluronic acid; P, treatment procedure performed at that visit

Safety

Expected transient post-procedural erythema resolving within the usual 48- to 72-hour recovery period was considered an anticipated local reaction rather than an adverse event. Two cases of prolonged erythema were recorded as adverse events: one in the microneedling-only group lasting nine days and one in the microneedling plus PLLA-HA group lasting seven days; both were mild, resolved spontaneously without intervention, and were not associated with edema, pain, crusting, desquamation, pigmentary changes, or infection. The safety population included all 30 participants across 90 full-face treatment sessions (45 per group). These findings reflect short-term tolerability and cannot establish the risk of uncommon or delayed PLLA-associated adverse events, including nodules or granulomatous reactions.

## Discussion

Results of this pilot comparative study suggest that microneedling combined with topical PLLA-HA may provide greater and more sustained improvements than microneedling alone. Benefits were observed across instrumental, imaging-based, and patient-reported outcomes and were maintained through week 12. The consistency of findings across different assessment methods strengthens the observed treatment signal, although the nonrandomized allocation limits causal interpretation.

Imaging findings generally paralleled the instrumental results, with greater improvements in wrinkles, pore visibility, and skin oiliness in the combination group. Treatment-specific satisfaction measures and GAIS also favored the combination treatment, whereas Satisfaction with Skin changed little, possibly because baseline scores were already high. Because participants and assessors were not blinded, subjective outcomes should be interpreted with appropriate caution.

These findings are consistent with preliminary evidence supporting the combined use of lactic-acid polymers and HA. Seo et al. evaluated combined PDLLA and non-cross-linked HA in 20 participants and reported significant improvements in elasticity, firmness, hydration, and overall skin quality after treatment [17]. These findings support the possibility that a lactic-acid polymer and HA may provide complementary effects. However, the product contained PDLLA rather than PLLA, was administered by injection rather than topical application, and the study did not include a microneedling-only comparator. Therefore, the reported within-group improvements do not establish superiority over microneedling, HA, or another active treatment.

Objective microneedling studies also explain why the monotherapy group in the present study showed some improvement. Wamsley et al. enrolled 35 participants, of whom 32 completed four microneedling treatments. Three months after the final treatment, facial elasticity had increased by 28.2% from baseline, while facial dermal and epidermal density increased by 101.86% and 19.28%, respectively; collagen type III and elastin expression were also significantly increased [18]. Ablon evaluated four microneedling sessions in 48 participants and reported statistically significant improvements in wrinkles, texture, and laxity, with the maximal response observed at day 150 [19]. These studies support a genuine remodeling effect of microneedling alone. However, because their principal analyses were within-group baseline-to-follow-up comparisons and they lacked parallel active controls, their p-values cannot determine the incremental benefit of adding a topical biostimulator.

Evidence involving HA and PLLA supports the possibility of complementary effects with different temporal profiles. Microneedle-assisted or intradermal HA-containing interventions have primarily reported improvements in skin hydration and superficial skin quality [20,21]. Prospective PLLA studies have described progressive clinical improvements over longer follow-up periods, consistent with the gradual effects associated with PLLA [22]. Clinical, ultrasound, and histological observations have also suggested that combining PLLA with high-molecular-weight HA may provide early improvements in hydration together with subsequent structural remodeling [23]. Additional evidence from stamp-type microneedle delivery of HA further supports its effects on skin hydration [24]. Collectively, these findings provide biological support for the combined approach, although differences in formulation, concentration, delivery depth, administration method, endpoints, and study design prevent direct comparison with the present topical protocol.

The temporal pattern observed in the present study is compatible with possible complementary effects of the combined protocol; however, the study was not designed to isolate the individual contributions of PLLA, HA, the reconstitution medium, massage, or altered device glide. Microneedling creates controlled epidermal and dermal microinjury and initiates wound-healing responses [18,19], while the resulting temporary microchannels provide a plausible route for microneedling-assisted topical delivery [2]. HA may contribute an earlier hygroscopic effect by increasing water retention and improving superficial skin quality [20,21,24], whereas PLLA may contribute more gradual collagen and extracellular matrix remodeling [5-7,23]. Both groups showed early improvement followed by continued changes over time; however, the combination group demonstrated larger and more persistent improvements through week 12. This early and sustained difference is compatible with an initial hydration-related contribution accompanied by progressive tissue remodeling, although dermal penetration, fibroblast activity, collagen synthesis, and histological changes were not directly assessed. Therefore, topical PLLA-HA delivered through microneedling-created channels should not be assumed to be pharmacologically or biologically equivalent to injectable PLLA or PDLLA.

Patient-reported outcomes were generally concordant with the instrumental and imaging findings. FACE-Q Satisfaction with Skin showed no significant within-group change, which may reflect a ceiling effect given the high baseline scores. In contrast, Satisfaction with Outcome and GAIS were more responsive and consistently differentiated the groups. A scale with restricted baseline variability may show little change even when objective measures improve, whereas a treatment-specific outcome scale may better capture perceived benefits. Nevertheless, the absence of participant and assessor blinding may have influenced subjective ratings.

Both interventions were well tolerated over the 12-week observation period in this small cohort. Mild, transient treatment-site erythema occurred in 1 of 15 participants (6.7%) in each group and resolved without treatment. No serious adverse events, infections, granulomas, or treatment discontinuations were reported. This pattern is consistent with the commonly described short-lived local reactions following microneedling and microneedle-assisted HA treatments [19,25]. Although these findings are reassuring, larger cohorts with longer follow-up would help further characterize uncommon, delayed, or product-specific adverse events associated with topical PLLA-HA delivery through microneedling-treated skin.

Several limitations should be considered. This was a single-center pilot study based on a clinic-derived non-probability sample, which limits generalizability. Although eligibility assessment and informed consent were completed by a non-treating investigator who was unaware of the upcoming assignment, the predefined alternating sequence remained predictable to the treating investigator and was not concealed; therefore, allocation-related selection bias cannot be completely excluded. Participants and assessors were not blinded. Baseline elasticity and hydration were imbalanced, and the pragmatic sample size was not based on a formal power calculation. Because the control group did not receive matched HA or reconstitution medium, equivalent massage, or product-assisted device glide, the observed between-group differences cannot be attributed specifically to PLLA or to any individual component of the combination protocol. Although Bonferroni adjustment was applied to selected repeated comparisons, the overall multiplicity arising from multiple outcomes, bilateral measurements, repeated visits, and different assessment methods was not fully controlled; therefore, the possibility of chance findings remains. Two week-12 assessments were missing; mixed-effects models used the available repeated observations, but missing values were not imputed. Home skincare and sun exposure were not objectively monitored, and the 12-week follow-up period may not capture the full duration of PLLA-related remodeling. Future studies should incorporate concealed randomization, blinded assessment, prespecified primary and secondary endpoints, adequately powered sample-size calculations, appropriate multiplicity-control strategies, and longer follow-up.

## Conclusions

Microneedling combined with PLLA-HA was associated with greater and more sustained short-term changes in selected skin-quality measures compared with microneedling alone, including instrumental outcomes, exploratory imaging measures, and selected patient-reported outcomes. The temporal pattern may be compatible with complementary effects within the combined treatment protocol; however, the present study was not designed to distinguish the individual contributions of PLLA, HA, or other procedural factors, and the underlying mechanisms were not directly assessed. Given the small sample size, non-randomized exploratory design, and baseline imbalances between groups, these findings should be considered preliminary and hypothesis-generating rather than definitive evidence of efficacy. Further adequately powered, randomized controlled studies with longer follow-up are required.
